# Organ-Specific Immune-Related Adverse Events for PD-1 Antibodies in Lung Cancer Treatment

**DOI:** 10.3389/fonc.2021.628243

**Published:** 2021-05-21

**Authors:** Xiaohu Zheng, Haiming Wei

**Affiliations:** ^1^ Division of Molecular Medicine, Hefei National Laboratory for Physical Sciences at Microscale, The CAS Key Laboratory of Innate Immunity and Chronic Disease, School of Life Sciences, University of Science and Technology of China, Hefei, China; ^2^ Institute of Immunology, University of Science and Technology of China, Hefei, China; ^3^ Research Unit Of NK Cells, Chinese Academy Of Medical Sciences, Hefei, China

**Keywords:** lung cancer, immune-related adverse events (irAE), PD-1 antibody therapy, inflammatory, side effect

## Abstract

Anti-PD-1 therapy has revolutionized the clinical treatment of lung cancer. With the increasing number of lung cancer patients being treated, there is also an increase in the number of immune-related adverse events (irAEs) being reported. These irAEs involve multiple organs and systems, mainly manifest as inflammatory side effects, and are different from the adverse events observed with traditional lung cancer treatment. These effects are often mild and treatable and reversible; however, in a few cases the side effects can be severe and lead to termination of immunotherapy. Management involves glucocorticoid-based related immunomodulators, which should be carefully prescribed to balance the efficacy and side effects of the PD-1 antibody treatment. This review will describe the characteristics and mechanisms of irAEs in specific organs, and will serve as a guide to help optimize treatment plans and improve patient outcomes.

## Introduction

Immunocheckpoint inhibitors (ICIs), especially PD-1 antibodies, have been a revolutionary success in the clinical treatment of tumors by blocking immune checkpoints to enhance anti-tumor immune responses. Normally, immune checkpoints include PD-1, which downregulates the T-cell response and serves to protect the body from potentially damaging immune responses. Tumors can hijack the system and evade the immune system by activating immune checkpoints and suppressing the T-cell response. Thus, interference with these immune checkpoint pathways can induce an anti-tumor immune response and deliver therapeutic benefits in cancer patients.

Several PD-1 antibodies have been approved by the United States Food and Drug Administration. Specifically, pembrolizumab and nivolumab were approved for the treatment of metastatic non-small-cell lung cancer (NSCLC). These antibody drugs have indeed shown significant efficacy in clinical trials. Programmed cell death 1 (PD-1) is a key molecule mediating immune tolerance in the body (1, 2). Blocking antibodies can definitely enhance the activity of the immune system, although this often results inflammatory side effects, which are referred to as immune-related adverse events (irAEs). The presence of irAEs has been reported in retrospective clinical trials evaluating PD-1 antibodies, which mainly included pembrolizumab and nivolumab, for the treatment of NSCLC (1–4).

**Figure 1:**
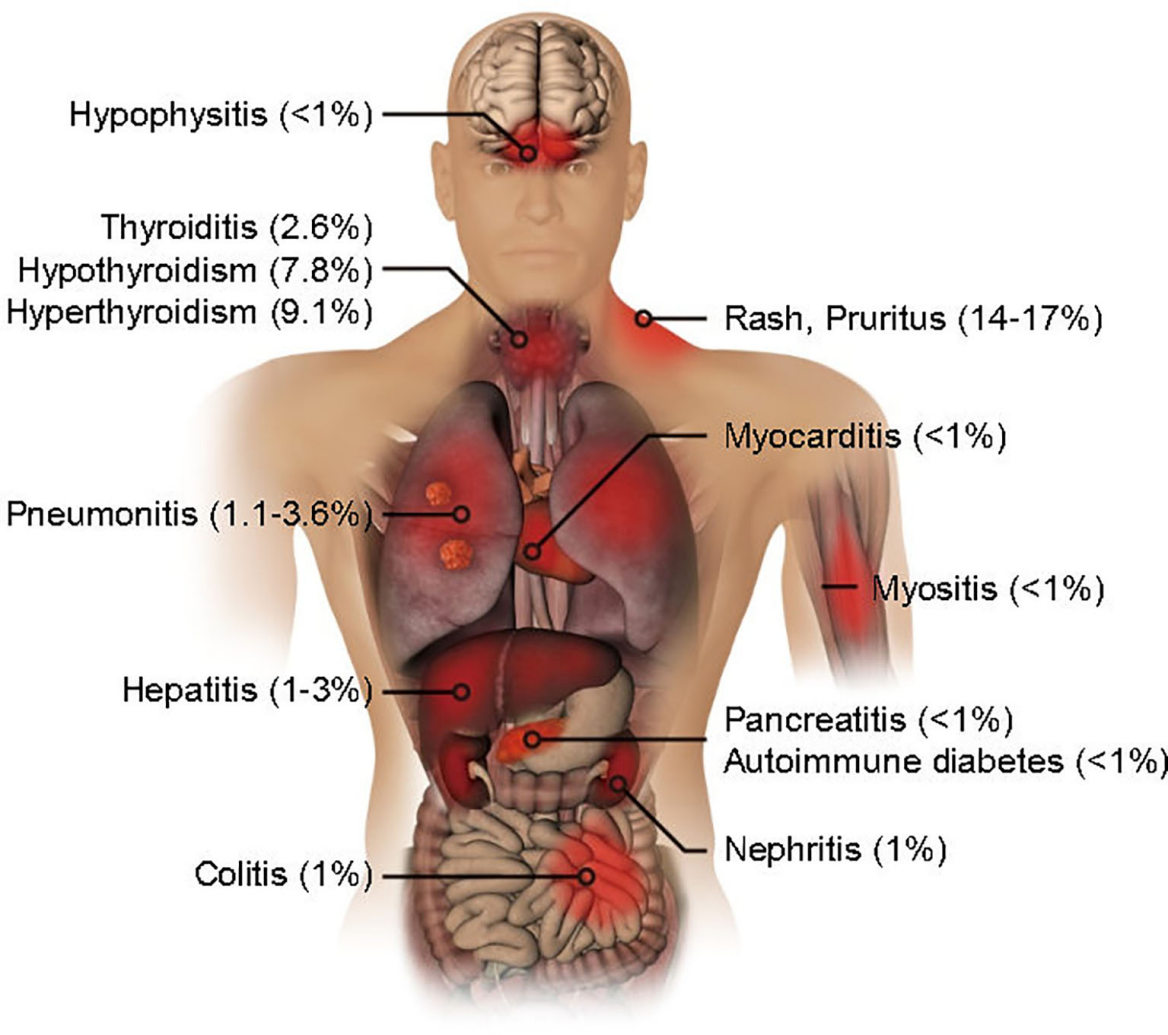
Organ-specific immune-related adverse events by PD-1 blockade in lung cancer treatment. The incidence rates are shown.

Clinical trial data suggest that the irAEs produced by PD-1 antibody in lung cancer treatment involve the thyroid, lung, skin, intestinal tract, and liver. Less common are the pancreas, kidney, pituitary gland, and musculoskeletal system (**Figure 1**). The majority of cases are mild irAEs and Anti-PD-1 therapy can usually be continued under close monitoring. Despite the very low incidence of moderate to severe irAEs, these may be associated with a serious decline in unique organ function and quality of life (5–9). Therefore, these toxicities require early detection and appropriate management. In this review, we focus on the pathological features, potential pathogenic mechanisms, and associated outcomes of irAEs in each unique organ, which is conducive to a more rational clinical management of lung cancer patients receiving PD-1 antibody treatment.

## Thyroid Dysfunction

### Clinical Characteristics

Thyroid dysfunction is a common and clinically mild irAE and is an early event among lung cancer patients treated with PD-1 antibodies (10). Most patients with anti-PD-1 drug-induced thyroid dysfunction are asymptomatic or present with hypothyroidism, hyperthyroidism, or thyroiditis (4, 5, 7–9, 11–14). The overall incidence rates of hypothyroidism and hyperthyroidism are 9.1% and 7.8%, respectively, while thyroiditis has the lowest reported incidence (2.6%) among PD-L1-positive NSCLC patients treated with pembrolizumab monotherapy (15). Hyperthyroidism occurs shortly after the initiation of pembrolizumab treatment and presents at median after 32 days (10). The onset of hypothyroidism occur later, at median time of 98 days. Many patients who eventually develop hypothyroidism experience a brief period of asymptomatic hyperthyroidism before the onset of the disease. Hypothyroidism may be asymptomatic or mild, and continued immunotherapy should not be precluded (7, 8, 10).

### Therapeutic Management

Clinically, patients with thyroid dysfunction are routinely given long-term thyroid hormone replacement therapy (10). Patients reporting this irAE did not experience a significant recovery of thyroid function, although none of the patients required corticosteroids, β-blocker, or methimazole therapy. Patients with abnormal thyroid function test (TFT) do not need to delay or stop using pembrolizumab due to the clinical impact of the thyroid dysfunction (8, 10, 12, 13, 15).

### Association With Clinical Outcomes

There was no significant difference in baseline clinical characteristics between patients with thyroid dysfunction and those without thyroid dysfunction. Interestingly, pembrolizumab-treated NSCLC patients with thyroid dysfunction had significantly higher median OS rates than patients without thyroid dysfunction (10). Whether there is a specific mechanistic association between antithyroid immunity and antitumor immunity is unclear, and larger clinical trials involving higher patient volumes are needed to verify the association.

### Possible Mechanisms/Pathophysiology

During anti-PD-1 therapy, patients with anti-thyroid antibodies may develop thyroid dysfunction, whether or not these antibodies are present at baseline or are detected after treatment begins. In addition, many patients who eventually develop hypothyroidism experience a brief period of asymptomatic hyperthyroidism before the onset of the disease (10). In addition, to T-cell-mediated cellular immunity, anti-PD-1 therapy may also regulate humoral immunity or enhance the activity of pre-existing anti-thyroid antibodies. PD-1 plays an important role in maintaining tolerance, and Anti-PD-1 therapy may disrupt the immune system’s ability to attack what it is meant to protect (16). Although it is suspected that the destruction of self-tolerance leads to thyroid autoimmunity, the mechanism through which PD-1 blocking leads to such autoimmunity is not clear.

## Cutaneous Reactions

### Clinical Characteristics

Dermatologic toxicity is one of the most common irAEs reported in lung cancer patients treated with PD-1 antibodies. Dermatologic toxicity manifests in a variety of forms, and commonly includes rash, pruritus, dry skin, pruritus, and dermatitis acneiform (1, 2, 14, 17). Clinically, a rash is relatively common. Specific symptoms include plaques, papules, and erythematous macules, mainly distributed to the trunk and extremities, and rashes can also be associated with pruritus (18). Dermatologic toxicity often develops in the early days following a 2–5-week treatment with anti-PD-1 blockage therapy. Dermatologic irAEs have been reported to occur in 14% to 17% of patients treated with nivolumab and pembrolizumab (7–9, 12–14, 19). Clinically, diagnosis is usually achieved by physical examination to assess the skin appearance, while skin biopsies are performed based on the dermatologist’s clinical diagnosis to define the cause (20).

### Therapeutic Management

Severe skin irAEs (grade 3–5 severity, according to the CTCAE) occur in only 1–10% of lung patients receiving PD-1 antibody therapy. Although this may vary according to clinical severity, for most patients topical corticosteroids are sufficient for treatment of rashes caused by immunotherapy (2).

### Association With Clinical Outcomes

The association observed between pembrolizumab treatment and irAEs has clinical relevance because the systemic side effects of pembrolizumab can act as a proxy for therapeutic response, similar to rashes treated with EGFR tyrosine kinase inhibitors (21). A previous meta-analysis of patients with advanced melanoma who had received PD-1 antibody immunotherapy found that the risk of death in patients with vitiligo was significantly lower than in patients without vitiligo (22, 23). As in the case of interleukin (IL)-2, dermatologic AEs resulting from targeted therapy are often associated with higher response rates, efficacy, and survival (24–26). Clinical data have suggested that dermatologic AEs are associated with a favorable outcomes in patients treated with pembrolizumab (27). There is still insufficient clinical data to determine whether PD-1-antibody-induced irAEs are associated with a favorable outcome in lung cancer.

### Possible Mechanisms/Pathophysiology

On histological evaluation, patients treated with pembrolizumab often present with an interface dermatitis or lichenoid tissue reaction. This may be due to non-specific activation of T cells after PD-1 blockade, resulting in attacks on susceptible keratinocytes. Ipilimumab inhibits tumor cells from evasive immune responses by suppressing the immune checkpoint cytotoxic T lymphocyte antigen-4 (CTLA-4), which also triggers autoimmune damage in previously protected normal cells. A similar mechanism may result for nivolumab and pembrolizumab as these antibodies target another immune checkpoint, the PD-1 receptor (18, 28–30).

## Hepatitis

### Clinical Characteristics

Hepatitis has a prevalence of 1–3% among anti- PD-1 trials in lung cancer patients. The most common manifestation of hepatitis is an asymptomatic increase in transaminase levels, which only occurs in patients with very severe or chronic disease (1, 17). Monitoring of transaminase and bilirubin levels before initiation of treatment with immune checkpoint inhibitors (ICI) and after each dose is necessary for hepatitis screening. Individuals with abnormal liver enzymes should undergo additional tests to rule out viral causes or chronic disease-related liver dysfunction (1, 2, 12, 15). Abdominal computed tomography (CT) scans show that the severity of liver side effects varies. In mild cases, the liver appears normal. However, severe cases are characterized by hepatomegaly, weakened hepatic parenchyma, and periportal edema similar to acute hepatitis (31, 32).

Viral infections of the liver are a risk factor for inducing hepatitis in lung cancer patients treated with PD-1 antibodies. Patients with past exposure to a high viral load of hepatitis B virus (HBV) can develop hepatitis during or after PD-1 antibody therapy. Patients with HBV infection can trigger more severe transaminase elevations (grade 3 or higher) (33).

### Therapeutic Management and Association With Clinical Outcomes

With the possibility of clinical therapeutic benefit and no remarkably increased risk, patients with hepatitis can choose continuous PD-1 antibody therapy. All events were of grade 3-4 and were subsequently treated with glucocorticoid or checkpoint inhibitor treatment was interrupted.

Data from case reports and phase II trials suggest that ICIs achieve a durable response and manageable safety in patients with controlled HBV or hepatitis C virus (HCV) infection (34). When treating patients presenting a history of hepatitis infection with ICI, regular monitoring of the status of the hepatitis virus is needed. Prospective studies are still needed to determine the true safety of ICI for the treatment of patients with viral hepatitis.

### Possible Mechanisms/Pathophysiology

The liver is an immune-tolerant organ. PD-1 is a key molecule mediating immune tolerance of T cells. Any immunotherapy that blocks the PD-1 receptor is bound to break the immune tolerance microenvironment of the liver and induce hepatitis (1, 32). In patients with viral infection of the liver, the resting state of the virus may be disrupted, triggering viral activity and the onset of hepatitis (32, 33).

## Diarrhea or Colitis

### Clinical Characteristics

For the treatment of lung cancer with PD-1 inhibitors, diarrhea is one of the most common irAEs (8–12.5%). In contrast, colitis has been reported in 1% of patients (1, 4, 5, 7–9, 12–14). The typical diagnosis of colitis includes an assessment to exclude the cause of infection and CT images to identify the severity and extent of colitis and to exclude the possibility of intestinal perforation. When a diagnosis is obscure, endoscopy is helpful. It can be used to assess patients with severe, refractory, or recurrent colitis and can help exclude cytomegalovirus-associated colitis and other high-risk characteristics (1, 35–37).

### Therapeutic Management and Association With Clinical Outcomes

Clinicians should first exclude infectious colitis in the differential diagnosis by obtaining the patient’s medical history, by examining physical appearance, or examining stool. For grade 1 symptoms, it is recommended to continue immunotherapy symptomatic treatment and close monitoring. For grade 2 symptoms, antidiarrheal use and symptomatic treatment are recommended. For persistent grade 2 symptoms, systemic corticosteroids should be attempted. If grade 3 or 4 symptoms occur, immunotherapy is discontinued with corticosteroid treatment. A monoclonal antibody against tumor necrosis factor (infliximab) is recommended for exacerbated severe symptoms and has been shown to significantly improve symptoms (31, 38–40).

### Possible Mechanisms/Pathophysiology

The histopathological characteristics of PD-1 antibody-associated colitis are very similar. The most common type of injury was active colitis with crypt atrophy and increased apoptosis. On biopsy, the mucosal lesions were mainly manifested as a neutrophilic crypt microabscess and inflammation, crypt atrophy, and edema. Another pattern of injury observed involved lymphocytic colitis, where biopsies showed increased intraepithelial lymphocytes (IELs), superficial epithelial injury, and increased laminar mononuclear inflammatory cells (33, 41–43).

## Pneumonitis

### Clinical Characteristics

The incidence of pneumonia at all levels in lung cancer was significantly higher than in other tumor types. One study reported that lung cancer patients treated with PD-1 inhibitors had significantly higher rates of full-grade interstitial lung disease (3.6% vs. 1.3%) and advanced interstitial lung disease (1.1% vs. 0.4%) than those treated with programmed death-ligand 1 (PD-L1) inhibitors (44). Pneumonia is first determined by checking oxygen saturation whilst ambulatory, and is then confirmed by CT images, determination of the infectious agent, and the degree of inflammation. CT images exhibit variable features, including interlobular septal thickening, cryptogenic tissue, ground-glass opacity, pneumonia-like or bronchiolitis-like appearance (44). The median time from the start of treatment to the onset of pneumonia was reported to be 2.6 months. The symptoms of most pneumonia patients include cough and dyspnea (45, 46).

### Therapeutic Management

As a clinical practice, PD-1 antibody therapy should not be terminated due to pneumonia. The vast majority of patients only need to receive corticosteroid treatment, and very few need to receive infliximab treatment. The prolonged time from the beginning of treatment to the onset of pneumonia (0.5 to 11.5 months) indicates that follow-up of signs and careful observation are important throughout therapy (11, 14, 46).

### Association With Clinical Outcomes

PD-1 inhibitor-associated pneumonia exhibits a range of imaging patterns that are associated with the level of toxicity. The safety evaluation of nivolumab in two phase I clinical trials reported pneumonitis-related death occurred in 3 cases (2.3%) (47) and in 1 case (1.1%) (48), respectively. The safety evaluation of pembrolizumab in two clinical trials reported pneumonitis-related death occurred in 3 cases (0.5%) (7) and in 1 case (0.2%) (3), respectively. A multidisciplinary approach exploring pulmonology, radiology, oncology, and pathology is required to optimize patient care.

### Possible Mechanisms/Pathophysiology

Lung cancer patients experience a higher incidence of pneumonia. The possible reasons are as follows: (1) the load of the primary lung tumor limits the stress and recovery capacity of the lung; and (2) these patients exhibit pulmonary fibrosis and chronic obstructive pulmonary disease.

## Nephritis

### Clinical Characteristics

In a systematic review of multiple randomized controlled trials of panitumumab and nivolumab for lung cancer, the incidence of nephritis was reported to be low (about 1%). Elevated serum creatinine levels was the most common characteristic of renal toxicity induced by ICIs (5, 9, 11, 12). A case of interstitial nephritis was reported in the nivolumab group receiving treatment for lung cancer (9). Pauci-immune glomerulonephritis also commonly presents as a renal injury. Generally, renal injuries occur during the later stages of PD-1 antibody therapy, that is, after 6–12 months of treatment (49).

### Therapeutic Management and Association With Clinical Outcomes

Most patients achieve complete relief with intravenous or oral steroid after 1–3 months. Very few patients require additional clinical hemodialysis (49).

### Possible Mechanisms/Pathophysiology

Histopathologic analysis of renal biopsies from cancer patients treated with nivolumab revealed mild, diffuse, active interstitial inflammation, mild edema, and tubular epithelial injury, consisting of abundant CD3^+^ and CD4^+^ T lymphocytes, and a small number of plasma cells, eosinophils and macrophages (49). In renal tissue, renal cells block the activity of PD-1 positive T cells by upregulating PD-L1 expression. Therefore, when PD-1 is blocked by antibodies, the PD-1/PD-L1 signaling pathway will also be blocked, and T cells will further proliferate and become activated, leading to cytotoxicity and kidney injury (49, 50). Thus, PD-1 antibody treatment may result in nephritis as a form of altered autoimmunity, similar to how autoimmune diabetes, may be based on the loss of peripheral tolerance of reactive T cells. Any situation that leads to an increase in T cell migration and function, may cause clinically significant kidney damage (49, 51, 52).

## Myositis

### Clinical Characteristics

Muscle injury mainly includes myalgia and myositis, and its typical symptoms include varying degrees of muscle weakness and pain. Less than 1% of lung cancer patients treated with PD-1 antibodies experience myositis, which is usually classified as mild (CTCAE grades 1 and 2). In general, the average onset time of myositis caused by immunotherapy is 25 days. Interestingly, ICI-associated myositis may manifest as classic muscle inflammatory symptoms, as well as ocular symptoms, similar to the autoimmune diseases observed at the neuromuscular junction (53).

### Therapeutic Management and Association With Clinical Outcomes

Of particular concern is that a high percentage of myositis occurs in association with myocarditis or myasthenia gravis, both of which cause a high percentage of deaths. Therefore, clinicians need to maintain a high index of suspicion and a low threshold for skeletal muscle biopsy results. Further, more systematic heart screening is required when myocarditis occurs simultaneously (53, 54).

### Possible Mechanisms/Pathophysiology

At present, most reports on myositis have not provided detailed clinical, immunological, and histopathological profiles, although a clinical trial study has shown that inflammation is the dominant feature and that most patients develop myositis-related autoantibodies, such as anti-muscarinic acetylcholine receptors (mAChR) antibodies (54–56).

## Hypophysitis

### Clinical Characteristics

Hypophysitis is an irAE that commonly presents following CTLA-4 antibody blockage but not with PD-1 inhibitor treatment (1). Symptoms of pituitary dysfunction are extensive, and include headache, weakness, visual changes, and enlargement of the pituitary gland (57). Pituitary inflammation induces secondary adrenal insufficiency, secondary adrenocorticotropic hormone (ACTH) deficiency, secondary hypothyroidism, and hypogonadotropin hypogonadism (1).

### Therapeutic Management

Several retrospective cohort studies have suggested that high doses of systemic corticosteroid therapy are not effective in reducing pituitary inflammation (58). Therefore, endocrine-related irAEs still require clinical exploration of more effective control methods, as long as immunotherapy is not terminated or the efficacy of antibodies is not affected.

### Association With Clinical Outcomes

A clinical study of CTLA-4 antibody in melanoma patients with hypophysitis suggested better antitumor efficacy was achieved (59).

### Possible Mechanisms/Pathophysiology

Some data suggest that pituitary inflammation may be associated with B-cell immunotoxicity and autoantibody production, including upregulation of anti-GNAL antibodies, or anti-ITM2B antibodies in patients with pituitary inflammation (1).

## Pancreatitis

### Clinical Characteristics

The pancreas is an organ rarely affected by PD-1 antibody treatment in lung cancer therapy. The clinical features of irAE-associated pancreatitis are varied and difficult to identify. Asymptomatic elevation of serum lipase and/or amylase levels during ICI treatment hampers the diagnostic process. During ICI therapy, serum lipase and/or amylase may be elevated, but the patient remains asymptomatic (60).

### Therapeutic Management and Association With Clinical Outcomes

The treatment of pancreatitis remains a difficult clinical problem, and immunotherapy may have to be suspended in due course. At present, the treatment of pancreatitis involves large doses of systemic glucocorticoids, and requires long-term administration, which gradually reduces patient symptoms and allows normalization of serum lipase levels. Delayed secondary pancreatic insufficiency may occur even after successful treatment, and patients must be regularly monitored (60).

### Possible Mechanisms/Pathophysiology

Pancreatitis is a rare immune-associated adverse event with PD-1 antibody treatment. Its imaging features are similar to those of autoimmune pancreatitis. Clinical evidence suggests that the pathologic characteristics of nivolumab in treating pancreatitis are similar to those of autoimmune pancreatitis (60, 61).

## Treatment of irAEs in Lung Cancer Treatment

Steroids and/or immunosuppressants are common clinical treatments for irAEs, and may this be associated with reduced efficacy of cancer immunotherapy. Given their immunosuppressive activity, the potential effects of glucocorticoids on the anticancer activity on inhibition of immune checkpoints must be considered. The results of multiple retrospective studies investigating melanoma are exciting (62). Steroid use was not associated with reduced efficacy of CTLA-4 inhibitors and PD-1 or PD-L1 inhibitors. Interestingly, patients exhibiting irAEs experienced a longer progression-free survival than patients without irAEs, and the benefits did not change with steroid use. Nonetheless, the use of prednisone during early treatment is associated with a poorer prognosis in lung cancer patients (63). Thus, prospective studies are still needed to determine the effects of steroid use on lung cancer outcomes in patients receiving PD-1 antibody therapy. These data suggest caution in the use of steroids or immunosuppressants.

In addition, low doses of corticosteroids can significantly impair the antitumor activity of T cells. Different organs also present different adverse effects (64). Therefore, additional clinical trials are needed to verify whether safer targeted drugs or antibody drugs are more feasible based on the organ-specific mechanisms associated with immune-related adverse events following treatment with ICIs. For now, treatments for moderate or severe irAEs in a timely manner is needed.

## Discussion

Inhibition of immune checkpoints, especially PD-1 blockade, represents an increasingly important strategy in cancer treatment. Overall, treatment with PD-1 antibodies is relatively safe for lung cancer, and most induced irAEs are clinically manageable (1, 15). Most toxic effects are reversible, except effects on the endocrine system may be long-lasting. Deaths from irAEs are rare, but myocarditis, pneumonia and colitis may likely trigger them. Therefore, attentive clinical monitoring and management is very important.

Here, we mainly review the irAEs in lung cancer treated with PD-1 antibody. Is there any difference with other types of cancer? Cutaneous malignancies (including melanoma, squamous cell carcinoma of the skin, and basal cell carcinoma) with treatment with PD-1 antibody have a high incidence of dermatitis as to 43%, and the incidence of head and neck cancer was also increased to 20%, both significantly higher than that of lung cancer patients. Patients with cutaneous malignancies were significantly more likely to develop dermatitis than patients with noncutaneous malignancy, including lung cancer (65). Pneumonitis is a relatively rare irAE in PD-1 therapy. The incidence of pneumonia was ~1% in melanoma and renal cell carcinoma patients receiving PD-1 inhibitor monotherapy, and rose to 3.1% in non-small cell lung cancer patients (66, 67). These data suggest that tumorigenetic organs may exhibit a higher frequency of irAEs. In general, there is no significant difference in the occurrence of irAEs among different types of tumors (1).

Currently, several PD-1 inhibitor drugs have been marketed, among which the most widely used are pembrolizumab and nivolumab (1). There is no a depth view of the difference in percentage of irAEs regarding different anti PD-1 therapies such as pembrolizumab and nivolumab. Shrujal et al. reported that organ specific irAEs were evaluated with 2993 patients in the investigational arm (pembrolizumab 1459, nivolumab 1534) (2). Among the 1459 patients exposed to pembrolizumab 1.1% had colitis, 0.2% had hepatitis, 3.1% had pneumonitis, 7.6% had hypothyroidism and 0.4% had hypophysitis. Among the 1534 patients exposed to nivolumab 0.3% had colitis, 0.0% had hepatitis, 2.2% had pneumonitis, 5.9% had hypothyroidism and 0.3% had hypophysitis (2). These data suggest organ specific irAEs are uncommon with the anti-PD-1 drugs. General irAEs are largely similar. The rates of pembrolizumab induced irAEs was slightly higher than that of nivolumab. The reason for this slight difference remains an open question.

Consistent with the different functions of immune checkpoints, the types of irAEs associated with monotherapy targeting the CTLA-4 or PD-1 pathways also differ (68). Typically, PD-1 inhibitors are better tolerated than CTLA-4 inhibitors. Grade 3 and 4 irAEs are more common in CTLA-4 inhibitors than in PD-1 inhibitors (69). Of note, colitis, rash and hypophysitis were more common with CTLA-4 inhibitors, whereas arthralgia, pneumonitis, vitiligo, and hypothyroidism were more common with PD-1 inhibitors (70). The exact biological explanation for the differences in organ selectivity and severity in irAEs with different ICIs is not fully understood. Theoretically, CTLA-4 blockade might induce larger T cell proliferation and also down-regulate regulatory T (Treg) cells, while PD-1 blockade only activates T cell clones in a small number of lesions (71).

There have been few studies on biomarkers for the risk of developing irAEs of immune checkpoint inhibitor therapy. Specific CD8+ T cells, Interleukin 17, eosinophil counts have been related to irAEs but not Set the threshold (72, 73). There are some preliminary clinical data suggesting that a family history of autoimmune diseases, previous viral infections, and known autoimmunotoxic drugs are also potential related risk factors (74, 75). It has recently been reported that irAEs were more frequent among patients with he preexisting antibodies (76). For example, skin reactions are more common in patients who already have rheumatoid factor than in patients who don’t (76). Thyroid dysfunction is more common in patients with pre-existing anti-thyroid antibodies. Suzuki et al. reported that 12 of 9869 cancer patients treated with nivolumab developed myasthenia gravis, 10 of whom had pre-existing acetylcholine receptor antibodies (77). Therefore, it is worth further investigation that pre-existing factor is associated with the development of irAEs.

## Author Contributions

HW and XZ conceived and conducted the project. HW supervised the project. XZ and HW wrote the paper. All authors contributed to the article and approved the submitted version.

## Funding

This work was supported by the Natural Science Foundation of China (Reference Numbers: 81872318), CAMS Innovation Fund for Medical Sciences (CIFMS, 2019-I2M-5-073) and the Strategic Priority Research Program of the Chinese Academy of Sciences (XDPB1002).

## Conflict of Interest

The authors declare that the research was conducted in the absence of any commercial or financial relationships that could be construed as a potential conflict of interest.
